# Predictors of Effort-Reward Imbalance Among Employees Providing Three Types of Long-Term Care Services in Japan

**DOI:** 10.1093/geroni/igaa057.594

**Published:** 2020-12-16

**Authors:** Ayumi Honda, Elizabeth Fauth, Yin Liu, Sumihisa Honda

**Affiliations:** 1 Nagasaki University Graduate School of Biomedical Sciences, Nagasaki, Japan; 2 Utah State University, Logan, Utah, United States

## Abstract

Increasingly, employees are leaving their jobs as long-term care workers in Japan. The purpose of this study was to identify predictive factors of effort-reward imbalance (ERI) among employees in long-term care, to better understand factors associated with excessive effort and reduced reward. This cross-sectional study included 944 participants providing three types of long-term care: home-based (n=201), community-based (n=128), and institutional (n=615). Multiple logistic regression analysis was used to identify factors associated with self-reported ERI, where higher ERI scores indicated greater work-related efforts and lower rewards. Key independent variables included type of occupation, employment status, position, daily working hours, job satisfaction, and annual income. Our results showed that low job satisfaction was the sole common factor associated with ERI in employees across all three types of long-term care. Other predictive factors for ERI differed by type of long-term care services. Working longer hours predicted ERI in community-based and institutional care employees, but not home-based care employees. For institutional care employees, being a care manager, holding a position of department head, having family-related stress were risk factors for ERI, suggesting that in this setting, the rewards of higher income and more prestige in leadership positions are offset by greater work-related demands. In conclusion, factors associated with ERI were both common and distinct among employees providing different types of long-term care services. Adjusting work demands and working hours, and identifying unique contributors to ERI within specific long-term care settings may help with job retention in these occupations.

